# Effectiveness of Waterpik^®^ for oral hygiene maintenance in orthodontic fixed appliance patients: A randomised controlled trial

**DOI:** 10.1177/14653125231173708

**Published:** 2023-05-19

**Authors:** Daniel Tyler, Jing Kang, Hock Hoe Goh

**Affiliations:** 1 Department of Orthodontics, Leeds Dental Institute, University of Leeds, Leeds, UK; 2 Department of in Biostatistics, University of Leeds, Leeds, UK; 3 Department of Orthodontics, York Hospital, York, UK

**Keywords:** orthodontics, fixed braces, fixed appliances, fixed orthodontics, oral hygiene, Waterpik^®^, oral irrigator, water jet, cleaning

## Abstract

**Objective:**

To establish whether the use of a WaterPik^®^ alongside a manual toothbrush (WaterPik^®^ + MTB) is more effective for maintaining oral hygiene compared to the use of a manual toothbrush alone (MTB) in patients wearing fixed orthodontic appliances.

**Design:**

A single-centre, two-arm, parallel-group, single-blind, randomised controlled clinical trial with a 1:1 allocation ratio.

**Setting:**

Orthodontic department at York Hospital, York Teaching Hospitals NHS Foundation Trust, UK.

**Participants:**

A total of 40 fit and well participants, aged 10–20 years, being treated with upper and lower fixed orthodontic appliances.

**Methods:**

Participants were randomly allocated, using stratified block randomisation, to the control group (MTB) or intervention group ‘(Waterpik^®^ + MTB)’. Plaque, gingival and interdental bleeding indices were recorded at baseline, 8 weeks, 32 weeks and 56 weeks. A generalised linear mixed model was used to assess differences between groups.

**Results:**

An interim analysis of results was performed with 40 patients recruited and 85% of data collected. The overall mean differences between the groups were as follows: plaque index = 0.199 (*P* = 0.88, 95% confidence interval [CI] −0.24 to 0.27); gingival index = −0.008 (*P* = 0.94, 95% CI −0.22 to 0.20); and interdental bleeding index = 5.60 (*P* = 0.563, 95% CI −13.22 to 24.42). No statistical difference between the two groups was found for any variable. The trial was stopped at this point.

**Conclusions:**

In terms of oral hygiene, our study did not find evidence to support the claim of benefit of using a Waterpik^®^ in addition to a manual toothbrush for patients wearing fixed orthodontic appliances.

## Introduction

It is widely accepted that it is more difficult to maintain good oral hygiene when wearing orthodontic appliances (Anuwongnukroh et al., 2017). Most patients will develop some degree of gingival inflammation during their fixed appliance treatment (Verrusio et al., 2018). Inflamed gingivae can be uncomfortable and lead to gingival hypertrophy (Pinto et al., 2017). Although the hypertrophy usually resolves after appliances have been removed (Zachrisson and Zachrisson, 1972), this can make the appliance and teeth more difficult to clean around. There is also a change in the microbiome of the mouth during fixed appliance therapy with an increase in periodontopathic (Naranjo et al., 2006) and cariogenic (Marda et al., 2018) bacteria. The oral microbiome has been shown to remain ‘abnormal’ even 2 years after removal of the appliance (Ghijselings et al., 2014).

As well as the effect on the periodontium, stagnating plaque around orthodontic appliances predisposes to decalcification. This can leave permanent, unsightly white or brown marks on the teeth that can progress to cavitation. The incidence of decalcification varies depending on the diagnostic techniques and criteria used, and a meta-analysis reported an incidence of 68.4% (Sundararaj et al., 2015).

Plaque removal using a toothbrush is widely recommended for oral hygiene when wearing fixed orthodontic appliances (ElShehaby et al., 2020). Adjunctive aids such as specific orthodontic brushes (Marçal et al., 2022), interdental brushes (Berglund and Small, 1990; Bock et al., 2010), dental floss (Zanatta et al., 2011) or Siwak (Al-Teen et al., 2006) have been described in the literature with generally unimpressive results.

The WaterPik^®^ is an oral hygiene aid that uses a pulsing jet of water to remove debris. The WaterPik^®^ has been on the market since the 1960s, with the tip as a small tube. In recent years, an orthodontic tip has entered the market, featuring a tapered brush through which the ejected water is delivered. If used appropriately, the orthodontic tip could mechanically remove plaque alongside the cleaning action of the pulsing water (Gorur et al., 2009). Existing evidence suggested that over 4 weeks, using a WaterPik^®^ with an orthodontic tip improves the plaque control of patients wearing fixed orthodontic appliances (Sharma et al., 2008). However, a course of fixed orthodontic treatment takes on average 24.9 months (Papageorgiou et al., 2017). A suitably designed randomised controlled trial (RCT) that assesses orthodontic patients for a longer period using a WaterPik^®^ with the orthodontic tip, would be a valuable addition to the current knowledge base.

### Specific objectives and null hypotheses

The aim of the present study was to establish whether the use of a WaterPik^®^ alongside a manual toothbrush (WaterPik^®^ + MTB) is more effective for maintaining oral hygiene compared to the use of a manual toothbrush alone (MTB) in patients wearing fixed orthodontic appliances.

Null hypotheses: the use of a WaterPik^®^ + MTB compared to using MTB alone does not lead to a statistically significant difference in the following: plaque levels; gingival health; the incidence of oral hygiene-related trauma; adherence with an oral hygiene regime; and satisfaction with an oral hygiene regime.

## Methods

### Trial design and any changes after trial commencement

This study was a single-centre, single-operator, two-arm, parallel-group, stratified, single-blind RCT trial with a 1:1 allocation ratio, conducted in the Department of Orthodontics at York Hospital, UK.

Changes after the commencement of the trial were as follows:

1. Due to the COVID-19 pandemic, there was a reduced pool of potential participants. For the first 33 participants, stratified block randomisation was carried out based on age, gender and baseline interdental bleeding index. It is standard practice using stratified block randomisation that once enough participants in an individual block have been recruited, recruitment to this block would cease. However, the decision was made to continue recruiting patients who met the inclusion criteria, even if their block was full, and randomise them by a single coin toss, to ensure that the sample size was achieved (see ‘Sample size calculation’ below).
2. An interim analysis of results was carried out due to issues with recruitment during the COVID-19 pandemic, and the ethical challenges associated with carrying out research in the already overstretched NHS. Following the analysis, the decision was made to stop the trial as there had been fewer dropouts than expected and the sample size had been reached. The decision was discussed with the trial sponsor and ethics committee. Any data collected between the initial interim analysis and the decision being made to stop the trial were included in this final analysis.

### Participants, eligibility criteria and setting

Potential participants for the trial were consecutive patients treated by a single Specialty Registrar in Orthodontics at York Hospital, UK. Patients treated were non–fee-paying patients who were treated under the UK National Health Service (NHS). The eligibility criteria are shown in Table 1. Patients who did not meet the eligibility criteria or were unwilling to provide consent to take part in the trial, were excluded.

**Table 1.** Eligibility criteria for participants.

Patient factors	Age 10–20 years
	Good general health
	Normal manual dexterity
	Normal periodontal health
	Not currently using prescription toothpaste
	Currently brushing teeth at least twice a day
	Not already using a WaterPik^®^
Orthodontic factors	Full upper and lower arch fixed orthodontic treatment
	Pre-adjusted edgewise appliances with American Orthodontics^®^ MBT prescription brackets (American Orthodontics Corporation, Sheboygan, WI, USA)
	Brackets, as opposed to bands, on all teeth apart frommolars, which could be bonded or banded
	Bonded with Transbond^®^ XT (3M Company, Maplewood, MN, USA).

### Interventions

The interventions provided for each group are shown in Table 2. The intervention group received a manual toothbrush together with a WaterPik^®^, whereas the control group received a manual toothbrush alone.

**Table 2.** The interventions provided to both groups.

Intervention	Same as the control group (below)	
	Waterpik^®^ Water Flosser Model WP-560 (Water Pik, Inc, Fort Collins, CO, USA) with four spare orthodontic tips, to change every 3 months.	
	Demonstrated to use with orthodontic tip around the brackets and between the teeth systematically, starting at a low pressure and increasing if they felt comfortable. Once a day, at night, for approximately 1 minute. Standard script used to give instructions which was provided to take home as an aide memoire.	
Control	30-min OHE appointment with a qualified DN with further training in OHE, before appliances being placed. Diet advice, demonstration of toothbrushing around fixed appliances.	
	Immediately after placement of fixed appliances, further oral hygiene instruction on models using a standard script. Script provided to take home as an aide memoire.	
	Oral hygiene aids provided for duration of trial. Participant instructed to only use these.	Toothbrush: Oral-B^®^ 1-2-3 Classic Care Manual Medium (Gillette Company LLC, Boston, MA, USA)
		Toothpaste: Colgate Triple Action^®^ (Colgate-Palmolive Company, New York, NY, USA) 1450 ppm fluoride.
		Mouthwash: Wisdom^®^ Fresh Effect Coolmint Mouthwash (Wisdom Toothbrushes Limited, Haverhill, UK) 225 ppm fluoride.

DN, dental nurse; OHE, oral health education.

### Outcomes

Participants were seen at baseline (T0), 8 weeks (T1), 32 weeks (T2) and 56 weeks (T3). At these time points, clinical indices to assess oral hygiene and gingival health were completed. The trial indices were performed by the same clinician.

The primary outcome was plaque level, measured using the Orthodontic Modification of Plaque Index (OMPI) (Williams et al., 1991) and Plaque Index (PI) (Silness and Loe, 1964). PI was scored at baseline, before fitting the appliances, and OMPI scored at follow-up visits. The lowest possible score for these indices is 0, where no plaque is present, and the highest possible score is 3.

Secondary outcome measures were gingival health, incidence of oral hygiene-related trauma, adherence with an oral hygiene regime and satisfaction with an oral hygiene regime. Gingival health was measured using the Gingival Index (GI) (Loe and Silness, 1963) and Interdental Bleeding Index (IBI) (Caton and Polson, 1985). The lowest possible score for the GI is 0, where no inflammation exists, and the highest possible score is 3. The lowest possible score for the IBI is 0%, where no bleeding exists, and the highest possible score is 100%.

The outcomes and the outcome measures used are shown in detail in Table 3.

**Table 3.** The outcomes and the outcome measures used in the trial.

Outcome	Outcome measure	Description
Plaque	Orthodontic Modification of Plaque Index (OMPI) (Williams et al., 1991) or Plaque Index (PI) (Silness and Loe, 1964)	Adaptation of the PI (Silness and Loe, 1964) for patients wearing fixed appliances. All teeth from first molar to first molar in both arches were painted with disclosing solution (TePe PlaqSearch™ Advanced Disclosing Solution; TePe Munhygienprodukter AB, Malmö, Sweden) the and patient rinsed with water. Clinical photographs were taken for assessment of intra-rater reliability. Four sites on each tooth (mesial, distal, gingival and incisal to the bracket) were scored 0–3. Scoring criteria are the taken from the PI and shown in Supplement 1. Mean score for mouth was calculated. At T0, PI (Silness and Loe, 1964) was used to measure the plaque coverage on six index teeth, one in each sextant of the mouth. The index teeth are the UR6, UR2, UL4, LL6, LL2 and LR4. This was used instead of OMPI because OMPI requires brackets to be in situ. If a UL4 or LR4 had been extracted, UL5 or LR5 was used instead. Mean score for mouth was calculated.
Gingival health	Gingival index (GI) (Loe and Silness, 1963)	All upper and lower permanent teeth from first molar to first molar. Periodontal probe gently inserted into the gingival crevice of four sites on each tooth (mesial, distal, buccal and lingual), and a score of 0–3 allocated to each surface. The scoring criterion are shown in Supplement 2. Mean score for mouth was calculated.
	Interdental Bleeding Index (IBI) (Caton and Polson, 1985)	Wooden interdental stick was used to depress each of the interdental papillae from first molar to first molar. The interdental stick was inserted buccally and the papillae depressed 1–2 mm four times. The presence, or lack, of bleeding within 15 Seconds was recorded and a percentage bleeding score calculated. Where a tooth was missing, or significantly displaced so much that an obvious papilla was not present (such as in the case of a palatally ectopic canine), the sites mesial and distal to the tooth were not recorded.
Trauma	Soft-tissue examination	Soft-tissue examination was performed at each appointment to assess for soft-tissue trauma secondary to oral hygiene regime.
Adherence to oral hygiene regime	Frequency of oral hygiene practices	Participants were given oral hygiene diaries to take home and complete for the duration of the trial. They were asked to complete the diary every time they cleaned their teeth, estimating in minutes for how long their teeth were cleaned, and state whether the WaterPik^®^ was used. These data were to be used to assess frequency of toothbrushing, time spent toothbrushing and frequency of WaterPik^®^ use.
Satisfaction with oral hygiene regime	Participant satisfaction measured with questionnaire	Participants were asked to complete a satisfaction questionnaire following their appointment at 8 weeks and 56 weeks. Two separate questionnaires were completed to avoid the control group being asked questions about using the WaterPik^®^. The intervention group answered all the questions on the control questionnaire, with additional questions regarding the WaterPik^®^. The questionnaires were adapted from those used a similar trial (Saini, 2016).

### Sample size calculation

To establish sample size, a power calculation was performed by the trial statistician (JK) based on the primary outcome OMPI. The significance level of the study was set at 5% (α = 0.05). The clinically significant effect size was set as 0.5 based on previous studies (Sreenivasan and Prasad, 2017). The standard deviation of OMPI found in similar studies is around 0.3 (Clerehugh et al., 1998); therefore, this figure was used. The intended power of the test was 0.9 (90%, β = 0.10).

The Power Analysis and Sample Size software (PASS, https://www.ncss.com/software/pass/) was used to calculate the required sample size. The minimum sample size for each group was calculated to be 7, so 14 participants in total were required. To account for dropouts over time, as well as balancing the sample size in the stratified blocks (eight blocks based on age, gender and baseline IBI, Supplement 3), we planned to recruit 20 patients into each group, 40 patients in total.

### Randomisation: Sequence generation, allocation concealment and implementation

#### Sequence generation

Participants were allocated using stratified block randomisation. The participants were stratified based on three prognostic characteristics: gender, age and IBI at baseline. It has been shown that female orthodontic patients report cleaning their teeth more often than male patients (Kudirkaite et al., 2016). Furthermore, female patients have been shown to have lower levels of plaque and have a greater level of knowledge of oral health (Furuta et al., 2011). Age was chosen as it has been shown that older orthodontic patients report cleaning their teeth more often than younger patients (Kudirkaite et al., 2016). As the inclusion criteria allowed for patients aged 10–20 years, participants were split in to ages <15 years and ⩾15 years. To attempt to distribute the participants based on their baseline oral hygiene evenly, participants were split into ⩽20% IBI or >20% IBI.

Using three prognostic characteristics resulted in eight blocks into which patients could be placed. These eight blocks are shown in Supplement 3. As the trial aimed to recruit 40 participants, it was planned that up to six participants in each block could be recruited. Once this number of participants had been reached, recruitment into that block would cease. However, after participant 33 was recruited, this was changed to a single coin toss due to the COVID-19 pandemic and a reduced pool of potential participants being available.

For each block, six sealed opaque envelopes were produced by the author DT. Inside each envelope was a paper slip which read either ‘Intervention’ or ‘Control’. There were three intervention and three control envelopes produced for each block. A folder was produced for each block and labelled with the block number and description. The six envelopes were placed inside each of the eight block folders. These were kept separately and thus concealed the allocation sequence from DT.

#### Concealment

For allocation of the participants, sealed envelopes containing either intervention or control were used.

#### Implementation

Patients were enrolled into the trial at the start of their visit by DT. At the end of the appointment to place the fixed appliances, DT then allocated the participant into one of the eight blocks and informed the dental nurse of which block they were in. DT then left the clinic and the dental nurse took out the relevant block folder. The patient was then asked to choose an envelope from the folder, and this was opened by the nurse, allocating them into intervention or control.

### Blinding

This was a single blind trial. The operator carrying out the indices (DT) was blinded to the patient allocation. The participants could not be blinded to their allocation. The participants were told that they must not tell DT whether they had a WaterPik^®^ or not, and this was also included on the oral health education information given to the patients.

### Statistical analysis

Statistical analysis was performed using IBM SPSS version 27 software (IBM Corp., Armonk, NY, USA). Summary descriptive statistics were produced to report demographic features and for each oral health variable.

A generalised linear mixed model was used to assess whether there was a difference between the intervention and control groups in terms of all outcomes (OMPI, GI and IBI), over time. A generalised linear mixed model is the ideal statistical model as it allows comparison of groups over time with non-normally distributed data and missing data (Ibrahim and Molenberghs, 2009).

An intraclass correlation coefficient (ICC) was generated, using a ‘two-way mixed’ model to assess intra-rater reliability in the measurement of the OMPI. The comparison was made between the clinical score and a repeated measurement calculated from the clinical photographs at a separate time. Reliability testing was not carried out on the GI or IBI because reliability studies for invasive gingival indices have shown that there is a trend for scores to worsen, possibly because the first examination increases the tendency for the gingiva to bleed the second time around (Poulsen, 1981). The GI has been shown to have a high level of intra- and inter-rater reliability after 4–6 hours (Shaw and Murray, 1977), but we did not feel it would be ethical to see school-age patients twice in the same school day for this purpose. Non-invasive alternatives are available, but have been found to be less reliable (Marks et al., 1993).

The incidence of trauma secondary to oral hygiene practice, adherence with an oral hygiene regime and satisfaction with an oral hygiene regime were analysed by a comparison of frequency between the two groups.

### Ethical considerations

Ethical approval was sought for the trial through the Integrated Research Application System (IRAS). This was approved by the Health Research Authority and Health and Care Research Wales (HRA & HCRW) on 23 August 2019. The IRAS project ID was 266235. The trial was registered with York Teaching Hospital NHS Foundation Trust who acted as the sponsor. The trial was registered with ClinicalTrials.gov Protocol Registration and Results system. The Protocol ID is NCT04604262.

## Results

### Participant flow

**Figure 1.**
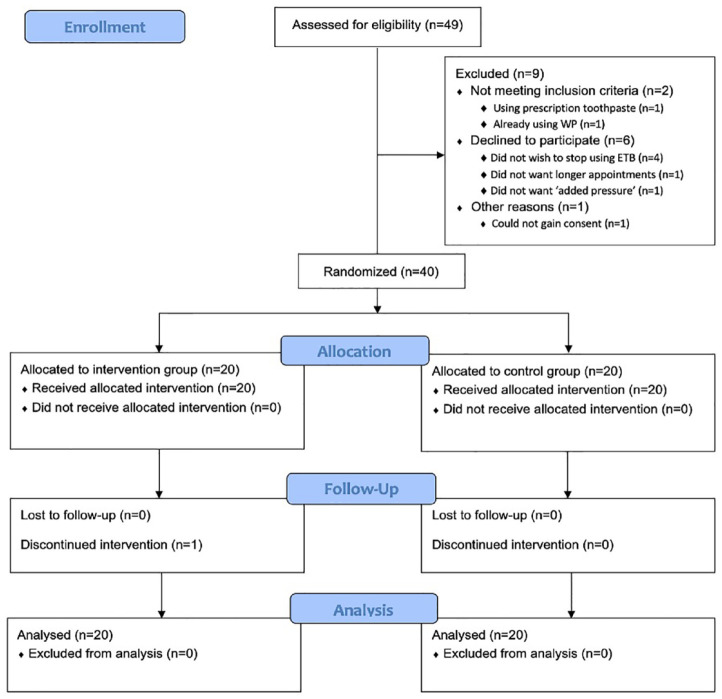
CONSORT participant flow chart. Adapted from Schulz et al. (2010).

A flow chart of participants through the trial is shown in Figure 1.

A single patient in the intervention group withdrew from the study after the 32-week indices, as he wished to start using an electric toothbrush.

Recruitment for the trial began on 15 November 2019. The first patient to be recruited started the trial on 6 January 2020. The trial was stopped early after the interim analysis of results on 2 November 2021. At the point of stopping the trial, 85% of data had been collected. A total of 40 participants had been recruited; all 40 participants had indices at 8 weeks (n = 20 intervention, n = 20 control); 32 participants had indices at 32 weeks (n = 17 intervention, n = 15 control); and 19 participants had indices at 56 weeks (n = 9 intervention, n = 10 control).

Fewer dropouts occurred than expected. At the interim analysis of results, the power calculation had been met for both groups at 56 weeks. Due to the constraints caused by the COVID-19 pandemic and extra pressure on the NHS, it was unethical to continue with the trial.

### Baseline data

Baseline data are shown in Table 4.

**Table 4.** Baseline data.

	Intervention	Control
Mean age at T0 (years)	14.9 ± 1.7	14.8 ± 1.6
Gender (% female)	50	65
Plaque Index	0.95 ± 0.3	0.94 ± 0.3
Gingival Index	0.77 ± 0.3	0.74 ± 0.2
Interdental Bleeding Index (%)	27.1 ± 24.8	34.7 ± 24.9

The groups were similar at baseline in terms of age. There were more female patients in the control group. The PI and GI were very similar at baseline between the two groups. The mean IBI was slightly higher in the control group.

### Numbers analysed for each outcome

The analysis was performed on an intention-to-treat basis. This means that all patients who were randomised to receive an intervention were included in the analysis, regardless of whether they completed the trial (Gupta, 2011).

The results for PI, GI and IBI are shown in Table 5.

**Table 5.** Difference of each outcome between control and treatment group using generalised linear mixed model.

	OMPI		GI		IBI	
	beta	95% CI	beta	95% CI	beta	95% CI
Difference	0.199	−0.24 to 0.27	−0.008	−0.22 to 0.20	5.6	−13.22 to 24.42

CI, confidence interval; GI, Gingival Index; IBI, Interdental Bleeding Index; OMPI, Orthodontic Modification of Plaque Index.

**Figure 2.**
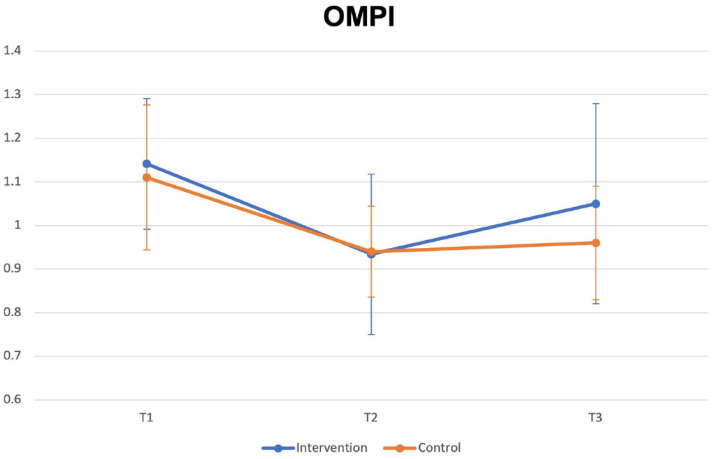
Mean OMPI at each time interval with 95% CI error bars. Baseline OMPI is not shown, as baseline indices were recorded before fitting appliances. The OMPI requires appliances to be in situ to be recorded. CI, confidence interval; OMPI, Orthodontic Modification of Plaque Index.

The mean OMPI at each interval with 95% confidence interval (CI) error bars is shown in Figure 2. The estimated difference between the treatment and control groups from the mixed model for OMPI was 0.199 (*P =* 0.88, 95% CI −0.24 to 0.27, R^2^ = 0.41).

**Figure 3.**
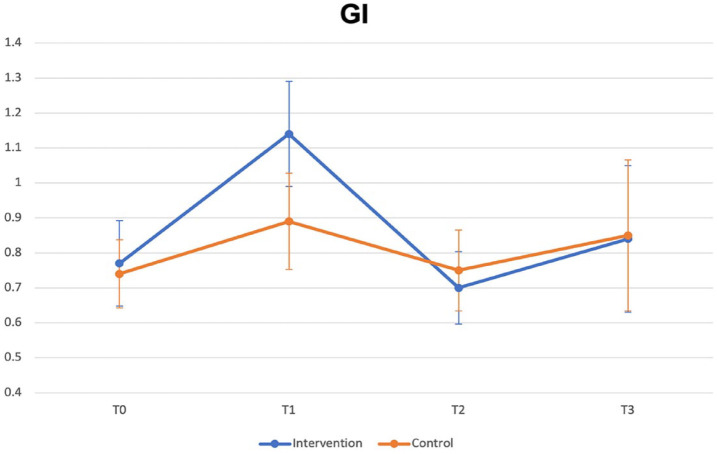
Mean GI at each time interval with 95% CI error bars. CI, confidence interval; GI, Gingival Index.

The mean GI at each interval with 95% CI error bars is shown in Figure 3. The estimated difference between treatment and control groups from the mixed model for GI was −0.008 (*P =* 0.94, 95% CI −0.22 to 0.20, R^2^ = 0.33).

**Figure 4.**
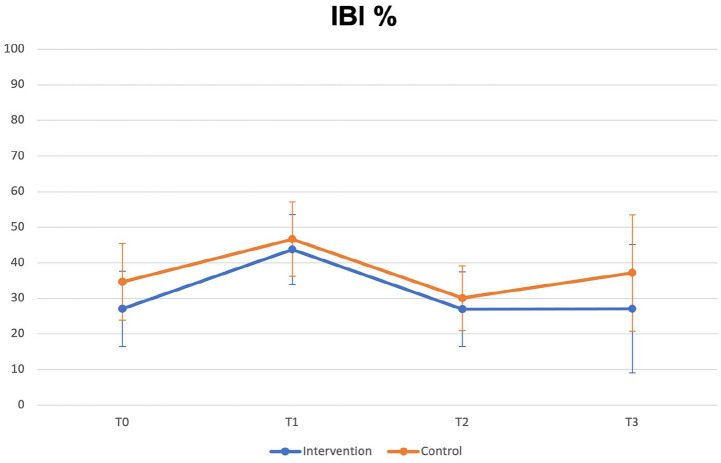
Mean IBI at each time interval with 95% CI error bars.

The mean IBI at each interval with 95% error bars is shown in Figure 4. The estimated difference between treatment and control groups from the mixed model for IBI was 5.6% (*P* = 0.563, 95% CI −13.22 to 24.42, R^2^ = 0.33).

The ICC for OMPI was 0.911 (95% CI 0.700–0.977), demonstrating good evidence for the repeatability of measurements of OMPI.

No participant had trauma detected at any visit in either group.

#### Adherence with oral hygiene regime

In terms of adherence with an oral hygiene regime, <25% of the oral hygiene diaries were returned, so it was not appropriate to analyse the data and draw any conclusions on adherence with prescribed oral hygiene regime. As a research group, it was thought that the data from those participants who did return the diaries would be biased to such an extent, that to present the data would be misleading.

#### Satisfaction with oral hygiene regime

Questionnaire responses regarding satisfaction with an oral hygiene regime are shown in Supplement 4. As the questionnaire was unvalidated, data are only presented for completeness. Due to journal word limits on reporting, data will not be analysed further.

### Harms

No harms were reported or detected for any participant.

## Discussion

### Interpretation

Based on the results of this trial, there is no benefit, in terms of plaque control or gingival health, in using a WaterPik^®^ for patients wearing fixed orthodontic appliances.

There is only one other published trial assessing orthodontic patients with a WaterPik^®^ with an orthodontic tip. Sharma et al. (2008) found a statistically significant difference in terms of plaque score over 28 days. However, plaque was measured using a different index in that trial: the Turesky modification of the Quigley and Hein plaque index (Turesky et al., 1970). This index scores from zero to five, assessing the facial and lingual surfaces of all teeth apart from third molars. Only participants with a score of at least 3.0 at baseline met the inclusion criteria. This means that only patients with sub-optimal oral hygiene at baseline were included, whereas participants with poor oral hygiene at baseline for this trial would have been excluded and unlikely to have been allowed to proceed with orthodontic treatment. The mean baseline plaque score was 3.73 out of a maximum score of 5 in the trial by Sharma et al., compared to 0.92 out of a maximum of 3 in this trial. Although the scores cannot be statistically compared due to the different methodologies, it does appear that the participants in the trial by Sharma et al. had worse oral hygiene. This RCT had a selection bias for patients who already have good oral hygiene.

Patients were recruited for the trial by Sharma et al. (2008) once they were already wearing braces, rather than being recruited before bond up. Therefore, the participants in the two trials were quite different. These differences could account for those in the results. Another potential reason for the differences found is the different lengths of follow-up. Sharma et al. (2008) followed up patients for a much shorter period. It may be that if the patients in this trial were seen after 28 days that a difference would have been seen due to the novelty value of the WaterPik^®^. It may be that over time as the novelty of the WaterPik^®^ wears off, patients use them less or less effectively. The novelty effect of the WaterPik^®^ has been acknowledged in other studies (Rosema et al., 2011).

### Limitations

There was a higher proportion of female participants in the control group (65%) compared to the intervention group (50%). As previously stated, female patients have been shown to have lower levels of plaque and have a greater level of knowledge of oral health (Furuta et al., 2011). Female teenagers have also been shown to be more likely to clean their teeth twice a day, compared to male teenagers (Currie et al., 2011). The presence of a higher proportion of female participants in the control group compared to the intervention group, could have masked the effects of the WaterPik^®^ in the intervention group; however, the differences are relatively small.

In terms of outcome measures, although the descriptors for scoring OMPI aim to be as objective as possible; it can be challenging to determine the subtle differences between whether a surface should be scored between a 1 and a 2 or a 2 and a 3. If a systematic error did indeed exist in the recording of the OMPI, this will likely have been applied to participants in both the intervention and control group. Therefore, it is unlikely to have affected the trial outcome.

Another issue with the OMPI is whether it is a valid surrogate measure for plaque control. It could be argued that the OMPI in this trial only represents the quality of plaque control before an orthodontic examination. The Hawthorne effect has also been shown to reduce tooth surface area covered with plaque in orthodontic patients (Feil et al., 2002). It is plausible that the participants were cleaning their teeth to a higher standard, both as a result of taking part in a trial and because they knew that they had an orthodontic appointment that day. However, again, these factors apply to both the intervention and control groups.

Like GI, photographs to assess the intra-rater reliability of the IBI were not feasible. However, the intra-rater reliability of this index has been found to be in the range of 91.3%–93.1% (Blieden et al., 1992). Research has shown it to be a more reliable clinical indicator of interdental gingival inflammation than other similar indices (Caton et al., 1988). The index has also shown to be valid, with histological investigation showing that bleeding sites are associated with histological changes associated with gingivitis compared to those that do not bleed (Bouwsma et al., 1988). The IBI is a relatively objective index; however, authors have suggested that although dichotomous indices are useful for patient education, for the purpose of research, quantitative measurements of bleeding are more appropriate (Panagakos, 2011). As with the GI, the possibility of false positives due to mechanical trauma also applies to the IBI, particularly due to the rigidity and shape of the wooden stick. However, it has been suggested that it may be more objective because there is less margin for variation in probe insertion depth, angulation or direction of movement (Hofer et al., 2011).

### Generalisability

The trial was carried out in a single NHS district general hospital and treatment was carried out by a single clinician. Patients in this setting do not pay for their treatment because it is funded by the NHS. It has been demonstrated that patients who self-fund orthodontic treatment have higher levels of compliance than those who receive state supported treatment (Wilson and Harris, 2015). Compliance was an important contributing factor to the outcomes of this study, and if the trial was carried out in a different setting, different conclusions may have been drawn. Furthermore, the malocclusions that are treated in secondary care settings are likely to be more complex than those treated in primary care (Jawad et al., 2015). Therefore, caution must be taken when generalising the results of this study to primary care or self-paying orthodontic patients.

## Conclusions

In the population studied, there was no benefit in terms of oral hygiene in the use of a WaterPik^®^ in addition to a manual toothbrush.

## Supplemental Material

sj-docx-1-joo-10.1177_14653125231173708 – Supplemental material for Effectiveness of Waterpik® for oral hygiene maintenance in orthodontic fixed appliance patients: A randomised controlled trial Click here for additional data file. Supplemental material, sj-docx-1-joo-10.1177_14653125231173708 for Effectiveness of Waterpik® for oral hygiene maintenance in orthodontic fixed appliance patients: A randomised controlled trial by Daniel Tyler, Jing Kang and Hock Hoe Goh in Journal of Orthodontics
