# Supplementary material for: Effectiveness of Waterpik® for oral hygiene maintenance in orthodontic fixed appliance patients: A randomised controlled trial
Source: J Orthod. 2023 May 19;50(4):367–77. doi: 10.1177/14653125231173708 (PMC10693741; doi:10.1177/14653125231173708)
Supplement: sj-docx-1-joo-10.1177_14653125231173708 – Supplemental material for Effectiveness of Waterpik® for oral hygiene maintenance in orthodontic fixed appliance patients: A randomised controlled trial [file sj-docx-1-joo-10.1177_14653125231173708.docx]

| Score | Description |
| --- | --- |
| 0 | No Plaque |
| 1 | A film of plaque adhering to the free gingival margin and adjacent area of the tooth. The plaque may be seen in situ only after application of disclosing solution or by using the probe on the tooth surface. |
| 2 | Moderate accumulation of soft deposits within the gingival pocket, or the tooth and gingival margin which can be seen with the naked eye. |
| 3 | Abundance of soft matter within the gingival pocket and/or on the tooth and gingival margin |

Supplement 1: Scoring criteria for the Silness and Loe Plaque Index (Silness and Loe, 1964). Where reference is made to the gingival pocket or margin, this was substituted with the bracket for mesial, distal and incisal areas.

| Score | Description |
| --- | --- |
| 0 | Absence of inflammation |
| 1 | Mild inflammation – slight change in colour and little change in texture |
| 2 | Moderate inflammation – moderate glazing, redness, oedema and hypertrophy. Bleeding on pressure. |
| 3 | Severe inflammation – marked redness and hypertrophy. Tendency to spontaneous bleeding. Ulceration. |

Supplement 2 Scoring criteria for the Gingival Index

(Loe and Silness, 1963)

| Block Number | Description | Planned Sample size | Recruited sample size | N Control | N Intervention |
| --- | --- | --- | --- | --- | --- |
| 1 | Female, <15 years old, ≤20% IBI | ≤6 | 9 | 6 | 3 |
| 2 | Female, <15 years old, >20% IBI | ≤6 | 8 | 4 | 4 |
| 3 | Female, ≥ 15 years old, ≤20% IBI | ≤6 | 2 |  | 2 |
| 4 | Female, ≥ 15 years old, >20% IBI | ≤6 | 4 | 3 | 1 |
| 5 | Male, <15 years old, ≤20% IBI | ≤6 | 4 |  | 4 |
| 6 | Male, <15 years old, >20% IBI | ≤6 | 5 | 3 | 2 |
| 7 | Male, ≥ 15 years old, ≤20% IBI | ≤6 | 3 | 2 | 1 |
| 8 | Male, ≥ 15 years old, >20% IBI | ≤6 | 5 | 2 | 3 |
| Total |  |  | 40 | 20 | 20 |

Supplement 3: Blocks used in the stratified block randomisation process.

| **Question** | **Possible Responses** | **8 weeks** | | **56 weeks** | |
| --- | --- | --- | --- | --- | --- |
|  |  | **Control** | **Intervention** | **Control** | **Intervention** |
| **‘How easy or difficult is it to use the Waterpik®?’** | Very easy  Easy  Neither easy nor difficult  Difficult  Very difficult |  | 40% (n=8)  50% (n=10)  5% (n=1)  5% (n=1) |  | 33.3% (n=3)  55.5% (n=5)  11.1% (n=1) |
| **‘When you clean your teeth, how often do you use the Waterpik*®*?’** | Every evening  Most evenings  Some evenings  Rarely  Never |  | 40% (n=8)  45% (n=9)  13% (n=3) |  | 33.3% (n=3)  44.4% (n=4)  11.1% (n=1)  11.1% (n=1) |
| **‘If you rarely or never use the Waterpik® we gave you, please tell us why’** | *Free text* |  | *n/a* |  | ‘*Stopped using it’* |
| **‘How clean or unclean do your teeth feel when you have used the Waterpik®?’** | Very clean  Clean  Neither clean nor unclean  Unclean  Very unclean |  | 30% (n=6)  55% (n=11)  10% (n=2)  5% (n=1) |  | 33.3% (n=3)  44.4% (n=4)  22.2% (n=2) |
| **‘How easy or difficult is it to clean your teeth with the manual toothbrush?’** | Very easy  Easy  Neither easy nor difficult  Difficult  Very difficult | 15% (n=3)  50% (n=10)  35% (n=7) | 30% (n=6)  50% (n=10)  15% (n=3)  5% (n=1) | 20% (n=2)  70% (n=7)  10% (n=1) | 33.3% (n=3)  22.2% (n=2)  33.3% (n=3)  11.1% (n=1) |
| **‘How often do you clean your teeth in the way that we have shown you?’** | Every time  Most of the time  Sometimes  Rarely  Never | 45% (n=9)  55% (n=11) | 45% (n=9)  55% (n=11) | 20% (n=2)  80% (n=8) | 44.4% (n=4)  55.5% (n=5) |
| **‘How helpful were the instructions we gave you about cleaning your teeth?’** | Very helpful  Helpful  Neither helpful nor unhelpful  Unhelpful  Very unhelpful | 35% (n=7)  60% (n=12)  5% (n=1) | 45% (n=9)  50% (n=10)  5% (n=1) | 40% (n=4)  60% (n=6) | 44.4% (n=4)  44.4% (n=4)  11.1% (n=1) |
| **‘If there is anything else you would like to tell us about brushing your teeth when you have braces, please write this below’** | *Free text* | *‘It’s hard to brush under the brace’* | *‘Hard to clean under brackets with manual’*  *‘It’s a bit awkward to get into nooks and crannies with manual’* | *‘Tricky not to knock the springs off’* | *‘Difficult to brush between brackets, but other than that, straightforward’*  *‘Hard to clean with manual’* |
|  | | n=20 | n=20 | n=10 | n=9 |

Supplement 4: Responses from satisfaction questionnaire. Responses to free text questions were very limited and are shown in full.
